# Supplementary material for: Expanding the Phenotype of the CACNA1C-Associated Neurological Disorders in Children: Systematic Literature Review and Description of a Novel Mutation
Source: Children (Basel). 2024 Apr 30;11(5):541. doi: 10.3390/children11050541 (PMC11119747; doi:10.3390/children11050541)
Supplement: Supplementary file 1 [file children-11-00541-s001.zip › children-2963101-supplementary.pdf]

CHECKLIST FOR CASE SERIES

Critical Appraisal tools for use in Systematic Reviews

	Ehtesham [11], 2022	Rodan [2], 2021	Chen [24], 2019	Bozarth [26], 2018	Quintela [27], 2017	Roberts [28], 2014
Were there clear criteria for inclusion in the case series?	Yes	Yes	Yes	Yes	Yes	Yes
Was the condition measured in a standard, reliable way for all participants included in the case series?	Yes	Yes	Yes	Yes	Unclear	Unclear
Were valid methods used for identification of the condition for all participants included in the case series?	Yes	Yes	Yes	Yes	Yes	Yes
Did the case series have consecutive inclusion of participants?	Yes	Yes	Unclear	No	No	No
Did the case series have complete inclusion of participants?	na	na	na	na	na	na
Was there clear reporting of the demographics of the participants in the study?	Yes	Yes	Yes	Yes	Yes	Yes
Was there clear reporting of clinical information of the participants?	Yes	Yes	Yes	Yes	Yes	Yes
Were the outcomes or follow up results of cases clearly reported?	Yes	Yes	Yes	Yes	Yes	Yes
Was there clear reporting of the presenting site(s)/clinic(s) demographic information?	Yes	Yes	Yes	Yes	Yes	Yes
Was statistical analysis appropriate?	na	na	na	na	na	na
